# Diamond Instruments

**Published:** 1881-01

**Authors:** 


*Diamond Instruments.*—The discovery that carbon points could be used for stone-cutting revolutionized that art, and rendered the boring of tunnels through granite mountains an easy task. It is safe to say that no advance in stone-cutting so great has been made since the gang-saw was invented. Carbon points for turning down hard steel rolls reduce these with an exactness and facility heretofore impossible. Most dentists are familiar with the "diamond disks" for separating, polishing, &c., and are aware how they take the place of the thick and fragile disks of corundum. Mr. S. Dessau, of New York, has made lately some very superior disks of this sort, and also some shapes hitherto unused. One of these he calls a diamond point. This is about the size of a silver five cent piece, and about the thickness of two nickels, and is the most useful cutting instrument of the kind we have had for a long time. For cutting down cusps of teeth that are in the way, for grinding off ragged edges, cutting down fillings, and especially for completing the articulation of artificial teeth at the final fitting in, it is indispensable; and it is hard to see how it has so far been done without. We had fancied that it would soon wear away and become smooth, but the copper seems *saturated* with the crystals, and after a month's use it cuts as well as ever. At the cost price, if this instrument only lasted a week, it would be cheap enough. A small and very neat diamond drill is also made, useful for forming cavities in artificial teeth, and for making depressions in their crowns in cases of difficult articulation. H.

---

